# Experiences of person-centred care - patients’ perceptions: qualitative study

**DOI:** 10.1186/1472-6955-13-28

**Published:** 2014-10-08

**Authors:** Tariq Saleem J Alharbi, Eric Carlström, Inger Ekman, Anders Jarneborn, Lars-Eric Olsson

**Affiliations:** 1 Institute of Health and Care Sciences, The Sahlgrenska Academy, University of Gothenburg, Box 457, SE 405 30 Gothenburg, Sweden; 2 Centre for Person-centred Care (GPCC), Gothenburg University, Gothenburg, Sweden; 3 Department of Medicine, Sahlgrenska University Hospital/Sahlgrenska, Gothenburg, Sweden

**Keywords:** Deductive content analysis, Health care models, Implementing care, Person-centred care, Patient-centred care, Patients’ experience

## Abstract

**Background:**

Patient care models have been implemented and documented worldwide. Many studies have focused on features that hinder and facilitate the shift to such models, including the implementation process, staff involvement, resistance to new models and cultural dimensions. However, few studies have identified the potential effects of such new care models from a patient perspective. The aim of the present study was to investigate whether patients did in fact perceive the intentions of partnership in the new care model 1 year after its implementation.

**Methods:**

Sixteen participants were interviewed, selected from two wards in a medical department where a new care model had been implemented 1 year earlier. A directed deductive content analysis was selected. The aim of the directed approach to content analysis was to investigate to what extent the new care model had been implemented, using patients’ perspectives to describe the level of implementation. A coding framework was developed based on a theoretical paper that described the key features of the new care model.

**Results:**

The implementation of person-centred care had clearly occurred to a large degree, even if some patients appeared not to have been exposed to the model at all. Aspects of the newly implemented care model were obvious; however, it was also clear that implementation was not complete. The analysis showed that patients felt listened to and that their own perception of the situation had been noted. Patients spontaneously expressed that they felt that the staff saw them as persons and did not solely focus on their disease. It was also stated that not every ailment or aspect of a patient’s illness needed to be addressed or resolved for open listening to be perceived as a positive experience.

**Conclusions:**

The findings indicate that even though some patients were not interested in participating and playing an active role in their own care, this might relate to a lack of understanding on how to invite them to do so and to increase their confidence. To change healthcare from a paternalistic system to care where patients are seen as partners may require pedagogical skills.

## Introduction

There has been a shift in focus on patients’ roles in healthcare. Person-centred care suggests that both patients and health professionals form a partnership rather than patients being passive receivers of care. Patient care models have been broadly implemented and documented [1-3] Furthermore, there are numerous studies on those features that can either hinder or facilitate the transition to such models. These can include the implementation process, staff involvement, resistance to new models and cultural dimensions [4]. However, few studies have tried to identify the potential effects of a new care model via patients’ perceptions [5,6].

## Background

The public healthcare industry has proven to be difficult to change. Although some studies from public hospitals have revealed a surprisingly strong culture of flexibility, the system is long standing and based on long and strong traditions [7-9]. Furthermore, several studies have revealed a high degree of inertia within several public healthcare organisations [10-12]. Similar to many counties health systems, the Swedish healthcare system is characterised by a Beveridge-like system, one that is largely publicly funded, non-marketed and difficult to customise. It was designed to collectivise and standardise the level of service [13,14]. This has proven to complicate efforts to tailor care to new care models that represent the modern imperative of patient involvement [15]. Authorities are calling for patients to play a greater role to improve their involvement in healthcare worldwide [16]. In the “Vienna Recommendations on Health Promoting Hospitals”, the World Health Organization [17] recognised the necessity of an active and participatory role for patients to improve both the quality and efficiency of the healthcare industry.

Several studies have been concerned with the need to involve patients in care processes. According to Longtin, the origin of patient involvement can be traced to movements in the 1960s that affirmed the consumer’s right to be informed, the right to choose and the right to be heard [1]. It has also been connected to ideas from New Public Management—ideals regarding market reforms and consumerism, that is, the freedom to choose one’s healthcare provider [18]. To enhance patient involvement, increase accessibility to the health industry and to strengthen the position of the patient, the Swedish government introduced a bill regarding changes in health and medical services [19,20]. A key aim was to guarantee access to healthcare staff who possess several types of competences and skills to ensure continuity and involvement in the care process. It was believed that this move would ensure a holistic approach to the needs of the patient and therefore establish a continuum during the care process [19,20].

However, the aim to implement a scheme to promote patient involvement is continually hindered by the paternalistic traditions within traditional healthcare systems [21]. Such behaviour relies entirely on the knowledge of experts and patients are regarded as passive recipients of care. They hold no legitimacy to be part of the imperatives of healthcare philosophies [22]. The replacement of out-dated care models with new models often stems from a desire to turn the patient from a passive receiver to an active key player [23]. The problem with the implementation of such schemes could perhaps be related to a lack of knowledge on how to involve patients in their care and the differences in preferences among people. Patients are more likely to trust their capacity to make decisions when they are thoroughly informed [24,25]. Say et al. found that many factors influence patients’ preferences in clinical decision making. Such factors include demographic variables, gender, experience of illness and medical care, diagnosis and health status, type of decision they need to make and their relationship with health professionals. They also found that patient preferences change over time, related to patients obtaining greater experience and the stage of their illness [26]. In a population-based study, Levinson et al. found similar results—while most patients wanted to be offered choices, only half wanted to leave the final decision to their physician. They also found that patients up to the age of 45 years had an increasing interest in being active in their care plans; this rate then declines with age [27].

### Impact of new caring models

Several studies concerning the implementation of new care models focus on staff perceptions and staff involvement and ignore patient involvement. Special involvement models have been developed to improve shared decision making exclusively between physicians and patients [28,29]. Involvement has also been facilitated by network groups of nursing staff, connecting different parts of the health service and guiding the patient through various organisations [30].

Some studies have measured patient satisfaction regarding new caring models that involve the patient and promote shared decision making. One study on a shared decision-making model found that patient satisfaction increased from 15.0% to 73.9% [4]. A study by Laugharne et al., regarding psychiatric care, found that a new care model involving patients was appreciated because the staff showed a personal touch that went beyond their expertise. However, not all patients experienced the impact of the new model [31]. Some still sought a greater shift in the balance of power between staff and patients, and others felt coerced and neglected by staff despite the new model. Similarly, in a study measuring the impact of patient-centred care, the results revealed that while some of the staff implemented the new care regime, others did not [32]. The conclusion in that study was that hospitals are missing relevant information when implementing such projects because patient surveys and interviews are seldom used in quality improvement work. A broad knowledge of the successes and shortcomings of implementation processes can provide the staff with information on how to handle likely obstacles [32].

Alharbi et al. found that even if managers agreed that person-centred care could be more effective, they differed regarding implementation strategies. Conflicting ideas from management contributed to confusion among staff, and thus the implementation process was delayed [33]. Attree et al. found that integration between organisations and different professions was a key factor in implementing new care models [30]. The concept of an inter-professional approach is often the focus when the aim is to involve the patient in implementing new patient-oriented care models [34]. Professional teams are often mentioned as a means to involve patients; however, suggestions on how to include the patient into the team are typically omitted [35]. Moreover, Légaré described how integrative concepts often lack interventions to support patient involvement [34]. Therefore, the study of an implementation has been focused on professionals and an organizational point of view, but not the patients who might reflect a different picture regarding the implementation and factors affecting it.

### The aim

The aim of the present study was to investigate whether patients did in fact perceive the intentions of partnership in the new care model 1 year after its implementation.

## Methods

### Setting

The decision to implement a new care model, the Gothenburg person-centred care model (gPCC), in a medical hospital clinic in western Sweden was made approximately 1 year prior to the patient interviews analysed in the present study. The decision was made based on the results of research studies conducted in the department where the gPCC was implemented and in another clinic [2,36,8]. The new care model marked a shift in the way staff viewed and collaborated with patients and their relatives [2]. More specifically, The gPCC model included a new style of admission, which lead to a health plan being developed within 24 hours from admission, which is based on the patient’s narrative at admission time. The theory behind the new care model was that, by focusing on the patient as a partner, the patient was empowered and therefore more active in the care planning [2]. In general, implementation processes are problematic and it is often difficult to know to what extent they have been successful [21]. One way to look for the effects of a new care model is to measure expected outcome results. In the new care model, the focus was on the patient as a partner, shared decision making and the documentation of a commonly agreed health plan.

### Data collection

Nineteen patients were selected from two wards in the medical department where the new care model had been implemented 1 year earlier. Inclusion criteria were patients with previous hospital experience who had received care for at least 2 days in one of the selected wards, able to understand and speak Swedish and who were lucid. Exclusion criteria were patients who did not want to participate or those judged as incapable of being interviewed because of mental impairment. The Ethics Committee in Gothenburg reviewed and approved the study (2011-10-04/T 825–11) and a permit was also obtained from the head of the clinic.

Participants were selected from two medical wards at a Swedish hospital. The patients who met the criteria were approached by a ward nurse and given both written and oral information about the study and its purpose and those who agreed to participate signed a consent form. The patients had a wide spectrum of internal medicine diagnoses and were between 22 and 91 years old (median 75 years, mean 67 years). Of the nineteen patients approached, two declined to participate. The interviews were conducted by one of the authors (AJ) during a four-week period and each interview lasted around 30 minutes. The interviewer used a semi structured interview guide with questions concerning the patients experiences of their role in the care; how they generally viewed patients role in health care; their relationship to the staff; if they perceived themselves as active or passive; how they perceived the information that they had been given during various stages of the care process, and how different phases were tailored to their individual circumstances.

One interview was excluded when it was found that the patient had been transferred from another ward where care planning and treatment had been completed. Because the person had not been subject to the ward routines, the interview was considered inadequate for the study. Hence, the analysis in the study was based on 16 interviews. Interview transcripts were coded electronically, using QSR NVivo 9 software.

### Analysis

A directed deductive content analysis was selected [37]. The aim of the directed approach to content analysis was to investigate to what extent the new care model had been implemented, using patients’ perspectives to describe the implementation level. This has been referred to as deductive category application [38]. The categories were based on an article that described the key features of the new care model [2]. In the present analysis, the key features became the main themes into which meaning units would be coded. The key features from the paper, together with an explanation on how the meaning units were coded into each category are presented at Table 1.

**Table 1:** Coding framework based on gPCC key features

***Code***	***Description***
*Initiating the partnership: patient narratives*	Meaning units were coded into this category when statements showed that health professionals listened to the patient’s narrative, dedicated enough time and interest to them, tried to understand the patient’s situation and identified resources and motivation. Patient narratives that showed that health professionals took interest in them as persons and looked beyond their illness were also coded into this category.
*Working the partnership: shared decision making*	Meaning units were coded into this category when statements described patients’ involvement in decision making and determining long-term and intermediate goals. Statements that described collaboration and discussions during the hospital stay were also coded in the shared decision-making theme.
*Safeguarding the partnership: documenting the narrative*	Meaning units were coded into this category when statements described the documentation efforts of patients and professionals. However, as the participants were patients and not health professionals, it was difficult for them to have real knowledge of the documentation. Meaning units were coded into this category when patients’ statements indicated they had been involved in planning care and care actions and/or aware of planned investigations and preliminary discharge plan ahead of time.

## Results

The analysis identified the key features of gPCC and produced six sub-categories. An overview of the results is presented in Table 2.

**Table 2:** An overview of the categories, sub-categories and examples of representative quotes

**Category**	**Sub-categories**	**Quotes**
***Initiating the partnership: patient narratives***	Being listened to	*“They are more interested in me as a person, in the personal stuff you know. Not just the disease.”*
	Not being listened to	*“They just have to check the computer to get my whole life.”*
***Working the partnership: shared decision making***	Being invited and involved	*“When I found out that my diabetes, that the values were high you know … I’ve been very clear about that they should teach me before I go home.”*
	Not invited to be involved	*“It’s the senior physician who decides it…well I have that, so that… it’s only to listen and take it.”*
	Not being invited but wanting to be involved	*“So any discharge date hasn’t been planned for? No they haven’t done that you see, but…Personally, I do think that I may be a few more days”.*
	Not wanting to be involved	*“I’m completely new at this whereas they are specialised in it, of course, so it becomes easy to rely on them.”*
***Safeguarding the partnership: documenting the narrative***		*“Yes, with the help of them, I can’t do it if I don’t have the documentation, hence that they have provided me with the tools in order to enable me to do so.*”

### Initiating the partnership: narratives

#### Being listened to

Some respondents noted that some health professionals were keen to know their personal situation (beyond the boundaries of the medical condition/hospital situation) when being interviewed for the first time, even though at that stage they were unsure of the relevance of such information. Furthermore, respondents highlighted that despite hospital environments and work routines being fast paced to deal with too many patients and not enough staff, they experienced what they thought was an uncommon practice: patients received the time they themselves deserved and wanted. One respondent described such an interaction with a physician in a positive way, appreciating the time and effort he was given, even though other doctors were not acting in the same way.

“*She seemed to take her time and listen, and not everyone does and sometimes you actually feel* “*oops what’s the hurry”.*

“They’ve came in, kind of one on one or kind of talked and that I appreciated.”

Furthermore, the respondents’ noticed how the health professionals asked for comprehensive information about their situation at home before hospitalisation. They focused on the respondents’ ability and what available resources they had to manage their illness after their discharge from hospital. One respondent described the experience during the first interview with a nurse and indirectly highlighted her interest in knowing his situation at home.

“At the very beginning they go through everything that’s happened during the time I was at home, so they’ve checked that up as best they could”.

Other respondents were surprised regarding the health professionals’ focus on them as people, and not simply focussing on the disease. Consequently, respondents were engaged in discussions with health professionals to try to better understand their illness. For example, one respondent described how health professionals in this ward used two-way communication that was not limited to the patient’s disease.

“We have talked…. We talked about personal stuff. I have answered mine and they have… I have asked them and they have answered theirs…They are more interested in me as a person, in the personal stuff you know. Not just the disease”.

#### Not being listened to

Not all patients were seen as partners; some described their experience of not being listened to, both intellectually and physically. Not being listened to intellectually encompassed situations where health professionals ignored what the patients had to say regarding their healthcare plan and treatment. More specifically, it was when health professionals talked among themselves about the patient’s current and future medical condition, directly in front of but not talking to the patient. This experience made them feel invisible. Another example of not being listened to included times when health professionals and patients did not communicate at the same level. More specifically, the doctor acted as an authoritarian figure and talked down to them. Some patients also described the dependence of medical technologies at the expense of actually talking to the patient. For example, there were instances when health professionals relied on data from a computer and rejected patient complaints about what they were experiencing.

“There’s this disappointment you get when they don’t believe you, when they took the blood gas, they said, but your blood tests look just fine…but why can’t I breathe!? So something is wrong. Well there’s nothing wrong with you was the answer I got.”

Furthermore, it was a common practice for health professionals to rely on computers to obtain patient information. The respondents noticed that health professionals would gather then divide the workload and finally approach patients with their equipment and begin to measure and investigate without even talking to the patient. Patients noticed that their input was unwanted and some patients thought their story was irrelevant. For example, one patient highlighted the extended dependence among health professionals on technology.

“They just have to check the computer to get my whole life”.

### Working the partnership: Involvement

#### Being invited and involved

Some respondents felt invited to participate and their desire to manage their illness after discharge was obvious. Such desire appeared common among those respondents’ diagnosed with a chronic disease. These respondents initiated discussions about their illness and requested to have both information and education from the health professionals to be able to manage the illness after discharge. For example, one respondent was diagnosed with a chronic disease and needed to be careful with her diet. For her, this was a big change in lifestyle and she was eager to find out everything she could about managing her illness

“When I found out that my diabetes, that the values were high you know … I’ve been very clear about that they should teach me before I go home. Now I’m going to see a dietician tomorrow.”.

The feeling of being involved and having partial responsibility for their illness provided a further sense of security and control of the future. One person disclosed that he had received tools to help him manage the disease himself. Several respondents stated that they felt a responsibility for their own body and health and therefore felt that it was important to be as active as possible during the care period. However, being informed did not necessarily mean to be involved. Several patients were well aware of the purpose of examinations and treatments. They felt that they were well informed, which generated a sense of security, tranquillity and of being well cared for. Sometimes this led to a tendency of passivity: they would let the staff deal with the care while they waited for the results. These patients did not express any notion of self-activity in the care process or participation in planning. Nor did they express any interest to do so or experience feelings of having been left out. They accepted the information given and the planning presented. They perceived their role as a patient to be passive and were comfortable with this. Several spoke of “orders” from physicians that they had to follow. They perceived, without exception, that doctors fully understood the overall situation and that the staff were capable of solving patients’ problems and this would result in the best outcomes.

“No they are working with this here now water and are going to try to remove it from my feet because it is so swollen. (Yes) that is what they’re doing [shows the IV] right, there you have that needle and that I drink and eat everything, that’s right.”.

#### Not invited to be involved

Several patients commented that they did not feel invited to participate and that they had not received sufficient opportunity to express themselves. Instead, the focus had been on short questions concerning the disease. The lack of personal contact and the feeling of not being seen as an individual lead to weaknesses in the relationships with the staff as well as concerns about medical issues. Questions arose regarding how well physicians can evaluate the effects of the medication if they are not aware of the person’s unique situation and characteristics. In some cases, concerns emerged as to what happens if a medicine does not work. Some patients felt that they were treated using a protocol of readymade prescriptions, without any regard to the individual. Other respondents highlighted that discharge decisions were made without their involvement and/or even knowledge. In such cases, when it was critical for some patients to be able to manage their illness after discharge, the lack of involvement by patients in the decision-making process to assess their ability to manage the illness by themselves at home led to a worsening health status. For example, one respondent described an experience during a previous care period where he underwent an orthopaedic procedure and was not involved in the discharge process. Discharge decisions were made by the health professionals based on their assumption that the patient was ready to go home. As a result, when the patient did return home, he was unable to manage everyday tasks, exposing him to unnecessary dangers. The way the discharge decision was made disappointed him, he thought they were wrong not to consult him.

“They thought I was fully treated so they thought I could cope on my own and at home. When going to the bathroom in the morning I spun around and fell down on the floor and I screamed, there was no one who could hear me, so there I lay for 3 hours.”.

In contrast with those respondents who accepted that health professionals have complete control over their healthcare plan, others felt unhappy about such things. Physicians’ dominance diminished respondents’ willingness to be involved and they became passive. For example, one respondent described how he felt when one of the medical consultants expected him to follow orders without any question.

“It’s the senior physician who decides it…well I have that, so that… it’s only to listen and take it.”.

However, some respondents stated that even though they themselves had not been invited to be involved in developing the health plan, other family members had. They made it clear that they were not at all happy about health professionals’ actions that excluded them from participating in developing their own health plan. The following quotes are examples of two respondents who felt left out when health professionals discussed their case with a family member.

“They talked to the children and not with me…they thought that the children knew what I wanted.”.

“It’s me they should ask how I feel not just decide this and that, luckily I had a younger son with me who later brought this up.”.

Not being properly listened to also caused rifts in the relationship between patient and staff. There was one example where a patient did not feel that she had been listened to and therefore did not feel that a health assessment by a physician was relevant to her. This person described an alternative explanatory model and did not trust the physician’s conclusions or instructions. Furthermore, she expressed reluctance about continuing treatments and follow-ups, which of course might cause problems for the care process.

*“Because you’re as a matter of fact in the hospital. You’re not here for the fun of it, no, but because it’s absolutely serious, you may have better contact with other physicians in the clinic than your own allocated physician… I think in that case there ought to be a little longer time to be present.”*.

#### Not being invited but wanting to be involved

In some cases, respondents felt completely left out and uninformed about decisions regarding their care and treatment goals. They felt this was important because they thought it could be difficult for them to manage their illness if discharged too early. These patients were concerned about their health status and wanted a long inpatient period, until they felt confident in managing their illness after discharge. For example, one respondent felt uncertain because he was unaware of his health plan and he wanted (and also expected) to stay in the hospital for a few more days.

“So any discharge date hasn’t been planned for? No they haven’t done that you see, but you don’t know, it could happen that they’ve done that. I don’t know. But I do doubt it. Personally, I do think that I may be a few more days. I think that but I don’t know.”.

#### Not wanting to be involved

Although some respondents were not invited to but wanted to participate, other respondents were unwilling to be involved in their healthcare. They held the perception that the doctor knew best and their involvement was not expected to lead to better treatment outcomes. Therefore, respondents described their relationship with health professionals as a vertical hierarchy where the patients were dependent on professionals. In the following quote, the respondent describes his hesitation to be involved. He attributed it to a perception about the supposed roles and responsibilities of health professionals and patients. As a result, the respondent played a passive role, relying solely on his health professionals.

“I kind of feel that I have to trust them. They know what medication I should take and what the plan should be like. I’m completely new at this whereas they are specialised in it, of course, so it becomes easy to rely on them.”.

### Safeguarding the partnership: documentation

Because the respondents were patients, it was difficult for them to determine staff efforts regarding documentation. However, the health plan was supposed to be shown to and discussed with the patient. It appeared that several respondents had been given the opportunity to discuss their health plan because they had a good understanding of what was being done and planned. They were able to absorb the information and had the opportunity to ask questions and reflect on their specific situation. An understanding of the proposed health plan was important for feelings of security. None of these patients were interested in exercising any influence medically but expressed a desire to have their personal preferences considered where appropriate. The reasons stated included for their own benefit and to maximise the possibility of a good outcome. Those who had objections or were totally opposed to a certain treatment or investigation were appreciative of how this was handled. They expressed the feeling of having been taken seriously, having been met with factual arguments and they ultimately felt respected for their decisions; they were not questioned or not made to feel guilty.

“I’ll say that I think it’s important that the patients themselves are active, yes, and not just say yes and amen, I think that is really important. And there are, maybe, I think, modern young patients are like that too, whereas it’s easier for us who are older to comply with the authoritativeness, yes, and say “yes of course doctor” but I think young people are completely different, yes, and that is good.”.

One respondent stated that he received, from his health professionals, the necessary tools to enable him to be involved in the planning of his care. These tools were simple: a pen, a few coloured markers and paper. Furthermore, when health professionals discussed the illness with the patient and possible influencing factors, the patient became motivated to take an active role to improve the outcomes of the healthcare plan.

“Yes, with the help of them, I can’t do it if I don’t have the documentation, hence that they have provided me with the tools in order to enable me to do so…using different pens, different colours. Helps them, and they administer various pills.”.

Some respondents noticed that the health plan also aimed to cover their requirements after the hospital stay. The health plan was meant to minimise or eliminate the possible negative impact of their illness on their everyday life. One respondent was informed about his health plan, which included a change in medication to make it easier to manage after discharge, as well as an extended recovery period to avoid aggravating his health condition.

“We switched to cortisone tablets from injections to make it easier for when I get home, I’m to go home…and tomorrow I’ll get to see the physician and get everything prescribed and, well yet another week on sick leave to rest and eat, I haven’t eaten very much. No, I’ve been on a drip…. I’ve received a good plan.”.

## Discussion

The main finding in the present study is that obvious effects stemming from the new care model were present in the data. However, its implementation was both fragmented and gradual; fragmented because not all staff appeared to be working according to the new care model (respondents perceived a difference between how individual staff members interacted with them) and gradual because the data indicated that staff who worked according to the person-centred care scheme did so in various degrees.

Traces of the implemented new care model were obvious; however, it was also clear that the implementation was not complete. The analysis gave the impression that a large portion of the staff understood and followed the instructions of the new care model to different degrees. Some conflicting data were also evident, indicating that some staff did not work according to the new care model at all. The analysis shows that patients felt listened to and that their own perceptions of the situation were noted. This was perceived positively and created trust. Patients felt secure in knowing that the healthcare professionals had listened to them and that their concerns had been taken seriously. Patients spontaneously expressed that they felt that the staff saw them as people and did not solely focus on their disease. It was also stated that not every ailment or aspect of a person’s illness needed to be addressed or resolved for open listening to be perceived as a positive encounter. To have the opportunity to express one’s thoughts and feelings and be listened to was, in many cases, considered to be the most important aspect. The fact that staff had a wider understanding of the patient’s situation provided peace of mind. In cases where the patient was already aware of his or her disease, it meant a lot to be taken seriously and to receive the help they sought. This confirmed a sense of personal responsibility for his/her situation and seemed to be a basis for a continuing relationship with their healthcare providers.

Two of the features described in the new care model, initiating the partnership and operating the partnership, were richer in data than the last feature, safeguarding the partnership. This may be explained by the results found in a parallel study on management strategies to implement person-centred care [33]. In that study, it was found that there was a lack of common strategies, each manager had their own thoughts and understanding of how to implement and operate the model. One of the aspects of person-centred care that all the managers agreed on, was that by understanding the patients better, care could be more effective and that money could be saved [33]. Many patients had previous care experiences and they sometimes compared these experiences with on-going care. They pointed out how both nurses and physicians showed more interest in them as persons and they did not just focus on the disease as they had done on previous occasions. They perceived this change as a positive addition that did not reduce the exiting level of medical care.

The interviews also revealed that respondents had different reactions to the new care. Some respondents felt that they were included and able to participate in their care, and they approved of the regime. In contrast, others felt they were not included but did not expect it. Of those respondents who were not included in their care, some did not mind and considered it natural to leave all decisions to the staff. They saw the staff as experts who were supposed to have that role; this is a common perception found in other studies [27]. Other respondents did not see it that way at all: they really wanted to participate although some felt excluded and not allowed to do so. Several respondents had experiences where the staff talked to each other over their heads, and in some cases staff discussed matters with the respondents’ relatives instead, which made the respondents feel belittled. Thus, it may not be that patients do not want to participate in their care but rather they have not been appropriately invited to do so. Eldh et al. found that if the discussion emanated from a patient perspective, patients were more likely to participate [25]. Furthermore, Say et al. concluded that health professionals should be more sensitive to patients as people to provide person-centred care [26]. In some cases, the respondents expressed uncertainty about openly talking about their situation and felt that their personal experiences were of no relevance to their medical situation. Great respect for physicians made it difficult to express feelings and personal thoughts. When such thoughts had been encouraged and their value affirmed, respondents rated the discussions as being very positive and considered it an important experience during their hospital stay.

The patient as a partner, as described in the theoretical paper by Ekman et al. was never intended to make medical decisions; instead, they can contribute to their care by being experts about themselves and by jointly guiding the care towards personally set goals [2]. However, some respondents were unclear about their role and how they could participate. This may have been unclear because in the previous study on the managers in this specific department, it was found they had different views on this issue, thus giving mixed messages [33]. However, one way to engage and involve patients is to start from a personal perspective, discussing their circumstances, pointing out factors influencing the outcome in connection to their health plan. Thus, respondents are drawn into the planning process and it becomes natural for them to participate.

### Limitations

Implementing a care model, such as gPCC, obviously includes a pedagogical challenge. One limitation of the study was the difficulty of identifying from patients’ narratives if or how staff tried to involve patients. The attitude among some of the excluded respondents was that the physicians would fix the problem and their own role was to follow physicians’ orders. A further limitation was the fact that it was difficult to determine from the interviews whether this was an effect of not being invited to participate or not wanting to be involved.

## Conclusion

The implementation of person-centred care clearly occurred to a large degree, despite some patients appearing not to have been exposed at all to the gPCC. These findings indicate that although some patients were not very interested in participating and playing an active role in their own care, this response might be because of a lack of understanding of how to invite them and to increase their confidence. Because of the existing paternalistic healthcare system in Sweden, it will require strong pedagogical skills to introduce new regimes where patients are seen as partners.

## Competing interests

The authors declare they have no competing interests.

## Authors’ contribution

Substantial contribution to conception and design: TA, L-EO, IE, EC, AJ. Acquisition of study data: AJ. Analysis and interpretation of data: TA, L-EO. Preparing, drafting and critically editing the manuscript: TA, L-EO, IE, EC, AJ. All authors read and approved the final manuscript.

## Pre-publication history

The pre-publication history for this paper can be accessed here:

http://www.biomedcentral.com/1472-6955/13/28/prepub
